# A Parameter-Adaptive Method for Primary Frequency Regulation of Grid-Forming Direct-Drive Wind Turbines

**DOI:** 10.3390/s24206651

**Published:** 2024-10-15

**Authors:** Siqi Hu, Keqilao Meng, Zikai Wu

**Affiliations:** 1 College of Energy and Power Engineering, Inner Mongolia University of Technology, Hohhot 010050, China; hannah_hu547@163.com (S.H.); 13932075138@163.com (Z.W.); 2 Key Laboratory of Wind Energy and Solar Energy, Ministry of Education, Hohhot 010050, China

**Keywords:** virtual synchronous generator, virtual inertia, damping coefficient, adaptive control, direct-drive permanent magnet wind turbines, primary frequency regulation

## Abstract

When wind turbines contribute to system frequency support using virtual synchronous generator (VSG) control, conventional VSG methods often fall short of meeting operational demands, particularly in terms of inertia and frequency support. In this study, considering both the frequency regulation and dynamic performance of VSG, a novel parameter design method that enhances frequency modulation capabilities is proposed in this paper. Initially, VSG control is integrated into the grid-side converter of a direct-drive permanent magnet synchronous generator (D-DPMSG) wind turbine. A small-signal model of the D-DPMSG-VSG active power is then formulated to analyze how the moment of inertia and damping coefficient impact system stability. Subsequently, ensuring that system parameter constraints are met, the key parameters of VSG are adaptively designed to dynamically adjust the system’s frequency and output power during transient responses. Finally, simulation results based on D-DPMSG-VSG in MATLAB/Simulink validated the feasibility, effectiveness, and advantages of the proposed parameter-adaptive VSG control strategy for enhancing the frequency modulation (FM) performance of wind turbines.

## 1. Introduction

On the background of dual-carbon goals, renewable energy generation, particularly solar and wind power, has garnered increasing attention from nations worldwide. As the capacity of grid-connected wind power continues to grow, its penetration rate within the power grid rises accordingly, introducing new challenges to the safe and stable operation of the power system [1]. On the one hand, the inherent randomness and intermittency of wind energy adversely impact the system’s frequency characteristics. On the other hand, the isolating effect of the wind power converter prevents the rotor dynamics of the wind turbine from coupling with the grid, thereby hindering participation in system frequency regulation and significantly compromising frequency stability [2]. The manner by which wind power participates in frequency regulation is one of the effective means for dealing with the friendly grid connection of wind power. Under an environment of high-permeability wind power, how to convert the frequency modulation characteristics of a synchronous generator in a wind power system to analog to enhance the stability of operational system has become a research hot spot.

Most wind turbines operate in maximum power point tracking (MPPT) mode. Under this mode, the unit wind turbine’s output power is unable to respond to grid frequency variations, leading to a reduction in the grid’s equivalent inertia and a subsequent decline in the voltage and frequency reliability at the grid connection point. To solve this issue, researchers have proposed employing VSG control, introducing virtual inertia and a damping coefficient to enhance power system stability. In refs. [3,4], VSG technology is applied to wind-storage hybrid systems to enhance the wind turbine’s primary frequency regulation capability and smooth its active output, thereby maintaining DC bus voltage stability. In refs. [5,6,7], VSG control is integrated with photovoltaic storage systems to regulate system frequency fluctuations, expediting the system’s response time and speed adjustment, thereby enhancing the grid-connected reliability of the photovoltaic system. The aforementioned studies rely on energy storage to facilitate new energy grid integration. However, incorporating energy storage increases construction costs and leads to relatively high maintenance expenses over time.

Some researchers integrate advanced algorithms with traditional VSG control to optimize performance. The authors of ref. [8] introduce a method that integrates an active disturbance rejection controller with VSG control to enable rapid frequency adjustments under large load disturbances. However, the inclusion of the active disturbance rejection controller complicates parameter tuning and yields suboptimal frequency regulation performance. The authors of refs. [9,10] combine fuzzy control with traditional VSG strategies to regulate frequency. Nevertheless, the determination of fuzzy rules and membership functions largely relies on experiential knowledge, lacking robust theoretical support. The authors of ref. [11] propose an adaptive VSG control method based on enhanced reinforcement learning (RL) to improve VSG frequency stability. However, the complexity of reward function design significantly increases the overall control complexity, with only small gains over traditional adaptive methods. The authors of refs. [12,13] propose a neural network-based adaptive control strategy for VSG parameter tuning, incorporating virtual inertia and dynamic damping compensation. The effectiveness of this approach heavily relies on the usability of representative training data, and inadequate data can significantly degrade performance. The authors of refs. [14,15,16] demonstrate that integrating model predictive control with VSG control significantly enhances system dynamic response speed and stability. However, in complex VSG systems, the optimization problems in model predictive control (MPC) algorithms can become highly intricate and time-consuming, particularly concerning online optimization solutions.

Contemporary studies on virtual synchronous generators mainly emphasize the tuning of adaptive control parameters and frequency optimization, proposing various enhanced control strategies to mitigate frequency deviations. The authors of ref. [17] introduce a differential correction term into the inertia damping control that alters the inertia damping characteristics of VSG across various frequency bands. However, the constant parameter VSG control method is still employed widely, although it lacks the capability to adjust dynamic parameters. Ref. [18] connects a switching delay link in parallel with the damping coefficient D to achieve decoupling of primary and secondary frequency modulation in VSG. However, the proposed improvement sacrifices dynamic response performance. Compared to traditional VSG secondary frequency modulation, this approach results in a longer frequency recovery time. The authors of ref. [19] explore VSG control strategies involving rotational inertia and damping compensation coefficients but do not consider the impact of the compensation amount on VSG stability. The effects on active power oscillation suppression and frequency modulation are also minimal. The authors of refs. [20,21] incorporate feedforward inertia and damping control into VSG control to enhance the system’s resistance to disturbances and suppress frequency and power fluctuations. The authors of ref. [22] propose combining fractional-order control (FOC) with VSG, validating its ability to suppress power and frequency oscillations in both islanded and grid-connected modes. However, the design approach for fractional-order controllers remains incomplete, with a notable absence of a general design methodology. The authors of refs. [23,24] integrate a proportional integral (PI) link into the power frequency controller to enhance frequency regulation capabilities. When the system load varies, control parameters require recalibration; otherwise, the system response speed will decrease. The authors of ref. [25] propose an effective transient damping power control strategy, which provides positive damping during transients, suppressing active power and frequency fluctuations. Moreover, it maintains the integrity of steady-state frequency droop characteristics. However, the article lacks further constraints on transient damping values, and an excessively high damping coefficient can result in prolonged response times.

The traditional constant parameter VSG control method lacks the capability to adjust dynamic parameters, limiting its effectiveness in stabilizing system frequency and power. As a result, power or frequency fluctuations may sometimes exceed acceptable limits, compromising the steady operation of the power system. The authors of ref. [26] leverage the dynamic energy of the wind turbine to enhance the inertia of the VSG and adaptively adjust the rotational inertia to bolster the frequency regulation capability of the wind power system. However, this overlooks the impact of the damping coefficient on frequency regulation performance. In ref. [27], the adaptive rotational inertia VSG is implemented in multi-machine parallel inverters, allowing for adaptation to changes in the response frequency with varying load conditions. Nonetheless, with the same frequency change rate, the rapid response performance is diminished. Refs. [28,29] propose an enhanced VSG strategy incorporating adaptive inertia and damping coefficients to augment the flexibility of the VSG. The findings indicate that the improved adaptive parameter control effectively mitigates VSG output power and frequency overshoot. However, both articles lack detailed methodologies for the design of key parameters. Refs. [30,31] propose a VSG control method that employs adaptive inertia. The analysis results substantiate that the VSG control strategy incorporating adaptive inertia exhibits superior stability and dynamic performance under load disturbances. However, the studies only provide a quantitative analysis of parameter changes without specifying the precise parameter value ranges. Ref. [32] suggests that the rotational inertia is maximized in the system’s steady state, with frequency adjustments achieved by adaptively reducing the rotational inertia to counter disturbances. Nonetheless, the study lacks simulation verification across multiple operating conditions.

Current research on VSG adaptive control predominantly emphasizes adaptive inertia control and detects the impact of VSG-related parameters on the dynamic performance of active power. However, it often neglects the influence on FM performance. The role of the VSG’s damping coefficient in FM scenarios is significant and cannot be overlooked. This paper addresses both FM and dynamic performance of the VSG by proposing specific design constraints for parameters. It enhances the FM capability of the grid-forming direct-drive wind turbine through the integration of VSG parameter-adaptive control, ensuring that the VSG not only meets FM requirements but also exhibits robust dynamic characteristics.

## 2. The Basic Principle of Wind Turbines Participating in Primary Frequency Regulation and the Control Strategy of Grid-Forming Direct-Drive Wind Turbines

According to the different energy sources of wind turbines participating in frequency regulation, the frequency regulation control of wind turbines can be divided into three categories: frequency regulation control based on reserved load shedding, frequency regulation control based on wind-storage combined systems, and frequency regulation control based on wind wheel rotational kinetic energy. For the frequency modulation control of wind turbines with the rotational kinetic energy of the wind turbine as the energy source, the wind turbine operates in the maximum power point tracking (MPPT) mode under normal conditions. Only when the frequency disturbance event occurs in the power grid does the wind turbine start frequency modulation control to release a part of the kinetic energy stored in the wind turbine, thereby increasing the output electromagnetic power to support the active power balance of the power grid.

**Figure 1:**
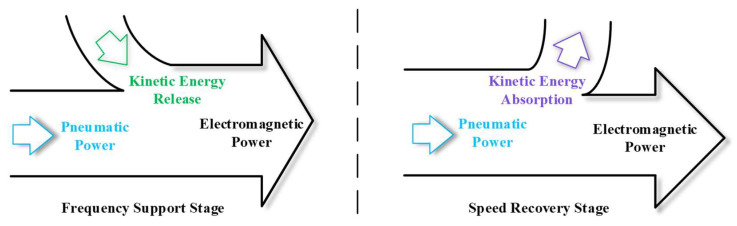
The release and absorption of wind wheel kinetic energy in the control of wind wheel kinetic energy frequency modulation.

This paper mainly focuses on the frequency modulation control of wind turbines based on the rotational kinetic energy of wind turbines (hereinafter referred to as the rotational kinetic energy frequency modulation control of wind turbine). As shown in Figure 1 below, the rotational kinetic energy frequency modulation control of a wind turbine first increases the output electromagnetic power by releasing the kinetic energy of the wind turbine (called the frequency support stage). At this time, the speed of the wind turbine decreases continuously with the release of the kinetic energy of the wind turbine. In order to avoid fan out of the operation due to the low speed, the wind turbine needs to reduce the output electromagnetic power in time to make it lower than the aerodynamic power, so as to make the speed rise again by absorbing the kinetic energy of the rotor (called the speed recovery stage) until the speed of the wind turbine returns to the normal operation state.

Common rotor kinetic energy control mainly includes virtual inertia control, droop control, and virtual synchronous machine technology. Based on virtual synchronous machine technology, this paper studies how to improve the frequency modulation performance of the power grid under the control of wind wheel kinetic energy frequency modulation through parameter optimization.

### 2.1. Dynamic Analysis of the Primary Frequency Regulation Process of Wind Turbines Based on the Rotational Kinetic Energy of a Wind Wheel

**Figure 2:**
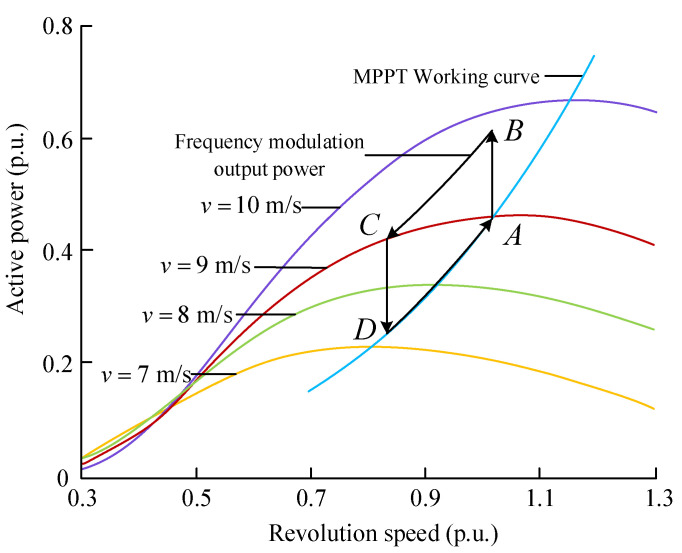
Schematic diagram of the primary frequency modulation process.

The schematic diagram of the primary frequency regulation process shown in Figure 2 clearly describes the dynamic process of wind turbines participating in power system frequency regulation. At a certain wind speed, the wind turbine is controlled by MPPT to operate stably at the maximum power point A, and the output power is controlled by AD. The control equation is shown in Equation (1). This control strategy can track the maximum power point more stably. When the power disturbance occurs in the power system and the wind turbine captures the system frequency drop signal, the output electric power is increased to point B by the auxiliary frequency control. At this time, the output electric power is greater than the input mechanical power, and the rotor begins to slow down to release a part of the kinetic energy. The change of speed also leads to a decrease of the mechanical power obtained by the wind turbine from the outside (as shown in the AC section of the figure). Therefore, in the primary frequency regulation process of the BC section, the total output power increment of the wind turbine is dynamically superimposed by the power released by the speed drop, and the power increment of the wind turbine is converted from the outside in real time.

Generally, the speed range of the wind turbine is 0.7~1.2 p.u. When the frequency modulation process ends, the speed recovery is performed according to the CDA. The speed recovery process also requires energy injection; otherwise, it may cause a secondary drop in frequency. The main purpose of this paper is to make better use of the ‘hidden‘ primary frequency modulation ability of the kinetic energy of the rotor. Here, the speed recovery control strategy is not studied in depth. The ratio of the speed to the wind speed of the PMSG wind turbine operating in the MPPT control mode is always the optimal speed ratio $\lambda_{\mathrm{opt}}$; that is, θ=0 and $\lambda =\lambda_{\mathrm{opt}}$. When there is no disturbance, the converter control output power $P_{we}$ and the generator rotor speed expressions are shown in the following formulas.

(1) $$\left\{\begin{array}{l} P_{we}=P_{MPPT}=k_{opt}{\left(\omega_{0}+\Delta \omega \right)}^{3}\\ \omega_{0}=\frac{\lambda_{opt}v}{K_{G}R} \end{array}\right.$$

Among these, $\omega_{0}$ is the optimal operating speed at different wind speeds, Δω is the change of speed, $k_{opt}$ is the MPPT control coefficient, $P_{MPPT}$ is the MPPT control output power, and $K_{G}$ is the gear ratio of the gearbox.

### 2.2. Basic Structure of Grid-Forming Direct-Drive Permanent Magnet Wind Turbines

**Figure 3:**
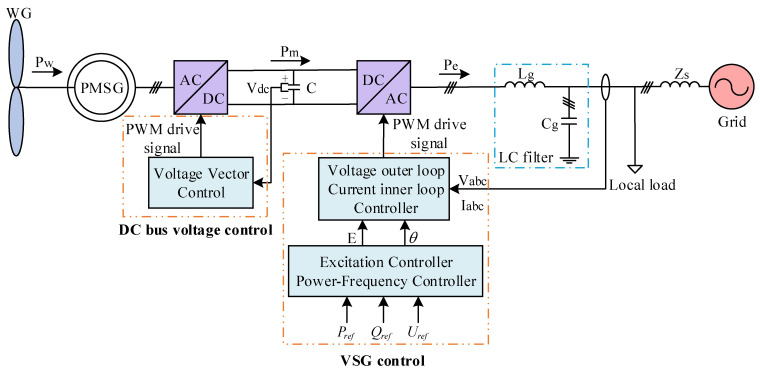
Basic structure of the grid-forming direct-drive permanent magnet wind turbine.

Figure 3 illustrates the permanent magnet direct-drive wind power system, which is based on voltage source VSG control. The system comprises a permanent magnet synchronous generator (PMSG), a bidirectional AC–DC converter, a DC bus, and a local load, among other components. Unlike traditional control structures, the generator side of the grid-forming direct-drive permanent magnet wind turbine employs voltage vector control to guarantee the reliability of the DC bus voltage. The grid side utilizes VSG control to adjust the power output of the wind power system, in which the power frequency control link manages active power, and the excitation link governs counteractive power. In the diagram, $P_{w}$ signifies the power formed by the wind turbine, $P_{m}$ denotes mechanical power, $P_{e}$ signifies electromagnetic power, $V_{\mathrm{dc}}$ indicates the DC bus voltage, and $V_{abc}$ and $I_{abc}$ correspond to the grid-connected terminal voltage and terminal current, respectively. $P_{ref}$, $Q_{ref}$, and $U_{ref}$ denote the reference values for active power, reactive power, and terminal voltage, respectively. $L_{g}$ represents the grid-side filter inductance, $C_{g}$ signifies the grid-side filter capacitance, and $Z_{s}$ denotes the grid impedance.

### 2.3. VSG Control Principle

**Figure 4:**
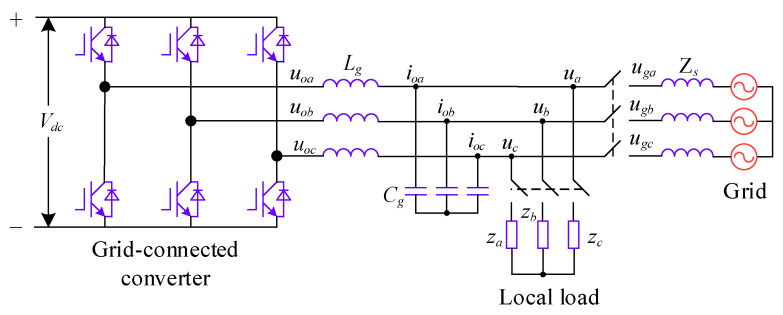
VSG main circuit topology.

Figure 4 illustrates the topology of the VSG’s main circuit, where $u_{oa}$, $u_{ob}$, and $u_{oc}$ represent the three-phase voltages output from the inverter’s bridge port; $u_{a}$, $u_{b}$, and $u_{c}$ denote the three-phase voltages produced by the VSG; and $z_{a}$, $z_{b}$, and $z_{c}$ correspond to the three-phase loads.

The VSG functions as a distributed power inverter utilizing indirect power control. It comprises a power frequency controller, an excitation controller, and a voltage outer loop with a current inner loop controller. During grid-connected operation, VSG control indirectly modulates the phase δ and reference voltage ($U_{ref}$) by adjusting the active power reference ($P_{ref}$) and reactive power reference ($Q_{ref}$). This regulates the active and reactive power infused into the grid indirectly.

**Figure 5:**
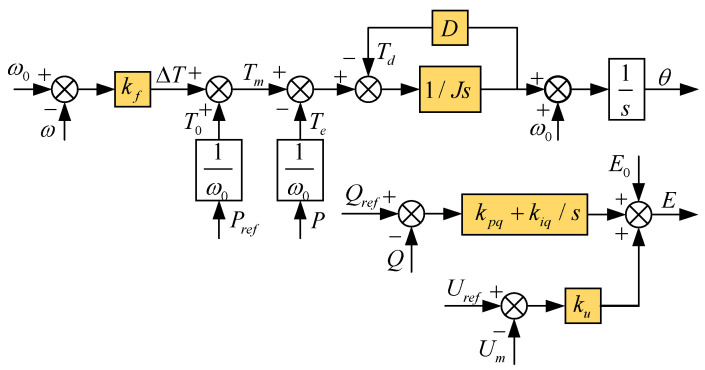
Power Frequency Controller and Excitation Controller.

Figure 5 shows the control block diagrams of the power frequency controller and the excitation controller.

The prime mover adjustment and rotor motion equations collectively form the power frequency regulator. The equations governing the rotor motion of the synchronous generator and the prime mover regulation are presented in Equation (2):

(2) $$\left\{\begin{array}{l} \begin{array}{l} J\frac{d\omega }{dt}=T_{m}-T_{e}-D\Delta \omega =\frac{P_{m}-P_{e}}{\omega }-D(\omega -\omega_{0})\\ \frac{d\delta }{dt}=\omega -\omega_{0} \end{array}\\ P_{ref}-P_{m}=k_{f}(\omega -\omega_{0}) \end{array}\right.$$

where $J\;(\mathrm{kg}\cdot m^{2})$ represents the virtual moment of inertia; D (N⋅m⋅s/rad) denotes the damping coefficient; ω and $\omega_{0}$ are the angular velocity and rated angular velocity, respectively; $T_{m}$ and $T_{e}$ are the mechanical and electromagnetic torques, respectively; $P_{m}$ and $P_{e}$ are the specified values of mechanical power and electromagnetic power; δ denotes the power angle of the system; and $k_{f}$ is the frequency modulation coefficient. To achieve precise control of reactive power by VSG during grid connection, the expression for the excitation controller is provided in Equation (3):

(3) $$E=E_{0}+(Q_{ref}-Q)(k_{pq}+\frac{k_{iq}}{s})+k_{u}(U_{ref}-U_{m})$$

where $U_{ref}$ and $U_{m}$ refer to the machine terminal reference voltage and the root mean square (RMS) value of the machine terminal voltage, respectively; $E_{0}$ is the no-load potential; $k_{u}$ is the voltage regulation coefficient; $k_{\mathrm{pq}}$ and $k_{\mathrm{iq}}$ are the proportional and integral coefficients of the reactive power loop PI controller, respectively; and Q represents the practical reactive power output by the VSG.

**Figure 6:**
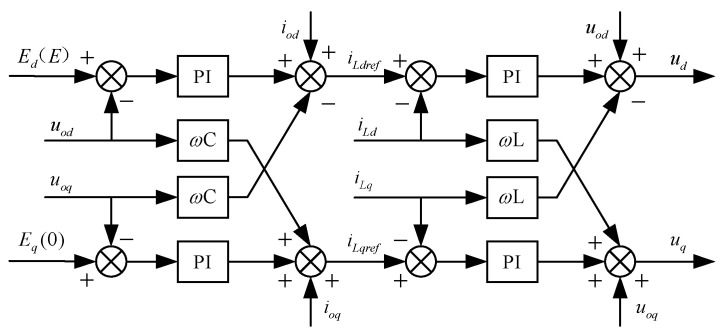
Double closed-loop controller.

The output voltage of the grid-connected inverter produces a three-phase AC command voltage-modulated waveform after the double closed-loop vector control of the voltage outer loop and current inner loop controller. Figure 6 presents the control block diagram of the voltage outer loop and current inner loop controller, which is based on an LC filter circuit.

In the figure, $E_{d}$ and $E_{q}$ represent the d-axis and q-axis components of the internal potential generated by the excitation controller; $u_{od}$ and $u_{oq}$ denote the d-axis and q-axis components of the output port voltage; $i_{od}$ and $i_{oq}$ signify the filter current and load current, respectively; $i_{Ldref}$, $i_{Lqref}$, $i_{Ld}$, and $i_{Lq}$ indicate the d-axis and q-axis components of the reference and actual values of the inductor current, respectively; and $u_{d}$ and $u_{q}$ are the voltage command values output by the double closed-loop controller. To simplify the control, it is advisable to set $E_{d}=E$ and $E_{q}=0$.

The core features of the dual closed-loop controller include high stability, fast response, high precision, flexibility, and scalability. This controller cooperates through two closed-loop control loops, so that the system can quickly adjust the control strategy and maintain the stability of the system when it is subject to external interference or internal parameter changes. In the process of parameter tuning of the controller, the parameters of the current inner loop controller are first tuned and then the parameters of the voltage outer loop controller are tuned. When considering the current inner loop to obtain faster current performance, the current regulator can be designed according to the typical type I system to improve the system performance [33]. Equation (4) is the calculation formula of the control parameters of the current inner loop PI regulator.

(4) $$\left\{\begin{array}{l} K_{ip}=\frac{R\tau_{i}}{3T_{s}K_{pwm}}\\ K_{iI}=\frac{K_{ip}}{\tau_{i}}=\frac{R}{3T_{s}K_{pwm}} \end{array}\right.$$

Since the main control function of the voltage outer loop is to stabilize the three-phase voltage, the anti-disturbance performance of the voltage outer loop should be considered when the control system is tuning. Obviously, the voltage regulator can be designed according to the typical type II system. Equation (5) defines the voltage loop PI regulator parameters.

(5) $$\left\{\begin{array}{l} T_{v}=5(\tau_{v}+3T_{s})\\ K_{v}=\frac{4C}{\left(\tau_{v}+3T\right)} \end{array}\right.$$

where $K_{ip}$ and $K_{iI}$ are the proportional regulation gain and integral regulation gain of the current inner loop, respectively. $T_{v}$ and $K_{v}$ are the proportional regulation gain and integral regulation gain of the voltage outer loop, respectively. R is the filter parasitic resistance; τ is the time constant corresponding to the pole of the open-loop transfer function of the typical system; $T_{s}$ is the current inner loop current sampling period; $K_{pwm}$ is the equivalent gain of the bridge PWM. The wind power system uses $K_{ip}=2$, $K_{iI}=25$; $T_{v}=2$, and $K_{v}=230$.

## 3. Design Principles for Key Parameters Considering FM Steady State and Dynamic Characteristics

According to Equations (2) and (3), the relationship between the frequency and active power of the power frequency controller can be obtained as Equation (6), where τ is the inertia time constant and d is the sag factor.

(6) $$\left\{\begin{array}{l} \frac{\omega_{0}-\omega }{P_{ref}-P}=-\frac{1}{(D\omega_{0}+k_{f})+J\omega_{0}s}=-\frac{d}{1+ts}\\ t=\frac{J\omega_{0}}{D\omega_{0}+k_{f}}\\ d=\frac{1}{D\omega_{0}+k_{f}} \end{array}\right.$$

**Figure 7:**
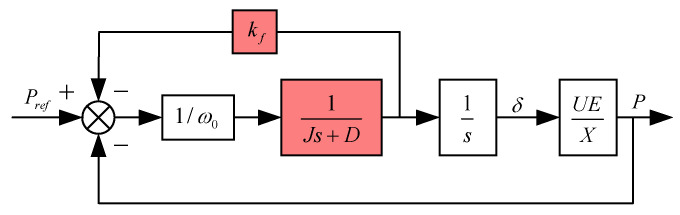
VSG active loop closed-loop control block diagram.

When the rotor motion equation is incorporated into VSG control, the transfer function becomes equivalent to a droop link with added inertia. J and D collectively determine the rate of change in the frequency response power, which reflects the system’s inertia. The droop coefficient (m) is influenced by both D and $k_{f}$. Higher values of D and $k_{f}$ result in a reduced frequency drop. When neglecting the effects of the active power measurement link and the coupling between active and reactive power, the active power control loop for the VSG power frequency regulation controller is illustrated in Figure 7. In this diagram, X represents the filter reactance.

From the above figure, the VSG active loop transfer function can be concluded as follows:

(7) $$G(s)=\frac{P(s)}{P_{ref}(s)}=\frac{\frac{1}{J\omega_{0}}\cdot \frac{EU}{X}}{s^{2}+(\frac{D}{J}+\frac{k_{f}}{J\omega_{0}})s+\frac{1}{J\omega_{0}}\frac{EU}{X}}=\frac{K}{J\omega_{0}s^{2}+(k_{f}+D\omega_{0})s+K}$$

where K=UE/X. The transfer function G(s) represents a typical second-order system. Provided the parameter design ensures power angle stability and the parameter design is reasonable, its output active power can achieve a zero-error response to the reference input. The parameters that dictate system performance include $k_{f}$, J, and D. From Equation (7), the undamped natural angular frequency $\omega_{n}$ and the damping ratio ξ of the second-order system can be derived as follows:

(8) $$\left\{\begin{array}{l} \omega_{n}=\sqrt{\frac{EU}{J\omega_{0}X}}\\ \xi =\frac{D}{2}\sqrt{\frac{\omega_{0}X}{JEU}}+\frac{k_{f}}{2}\sqrt{\frac{X}{J\omega_{0}EU}} \end{array}\right.$$

When ξ=0, the system behaves as a second-order undamped system; when 0<ξ<1, it is underdamped; when ξ=1, it is critically damped; and when ξ>1, it becomes overdamped. Considering 0<ξ<1 and a ±5% error band, the dynamic performance indices of this second-order system—overshoot σ%, peak time $t_{p}$, settling time $t_{s}$, and the number of oscillations N—are calculated as shown in Equation (9):

(9) $$\left\{\begin{array}{l} \sigma \%=e^{-\frac{\pi \xi }{\sqrt{1-\xi^{2}}}}\times 100\%\\ t_{p}=\frac{\pi }{\left(\omega_{0}\sqrt{1-\xi^{2}}\right)}\\ t_{s}=\frac{3.5}{\omega_{0}\xi }\\ N=\frac{1.5\sqrt{1-\xi^{2}}}{\xi \pi } \end{array}\right.$$

It can be seen from Equation (9) that the system’s overshoot is solely influenced by ξ. A smaller ξ results in a larger overshoot. Conversely, a larger ξ leads to a reduced overshoot in a second-order system, shorter settling time, and fewer oscillations.

**Figure 8:**
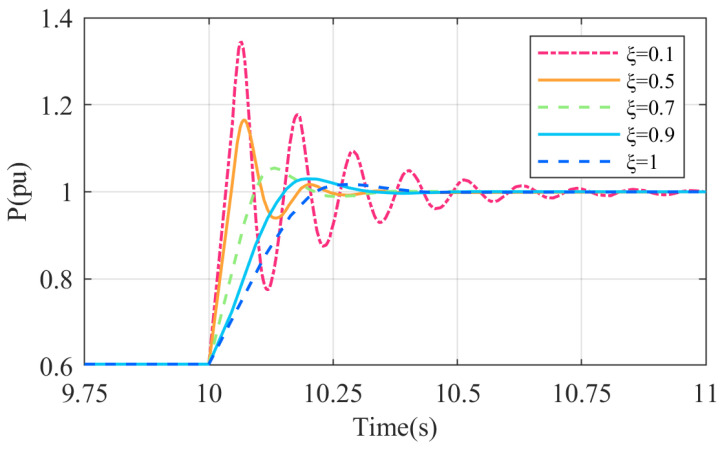
Active power jump response characteristics at different damping ratios.

An analogous model of the D-DPMSG-VSG system was developed employing MATLAB/Simulink. At t = 10 s, the wind speed increases abruptly from 10 to 13 m/s. Figure 8 illustrates the step reaction characteristics of the system’s output active power at various damping ratios.

According to the ‘second-order optimal’ system tuning method, the constraint condition for ξ is provided in Equation (10).

(10) $$\xi =\frac{1}{2}\sqrt{\frac{X}{J\omega_{0}UE}}(k_{f}+D\omega_{0})\approx 0.7071$$

When the grid frequency is stabilized at its rated value, the VSG responds to active power inputs without any deviation. As demonstrated by Equations (6) and (7), the VSG control exhibits primary frequency modulation capabilities due to its droop characteristics and can adjust abnormal frequencies when the grid frequency deviates from the rated value. The FM capability of the VSG control is influenced by d, which is governed by D and $k_{f}$. By combining Equations (6) and (10), it can be determined that the parameters of the VSG active power loop must meet the following conditions:

(11) $$\left\{\begin{array}{c} \frac{1}{d}=D\omega_{0}+k_{f}\\ d^{2}=\frac{X}{2J\omega_{0}UE} \end{array}\right.$$

**Figure 9:**
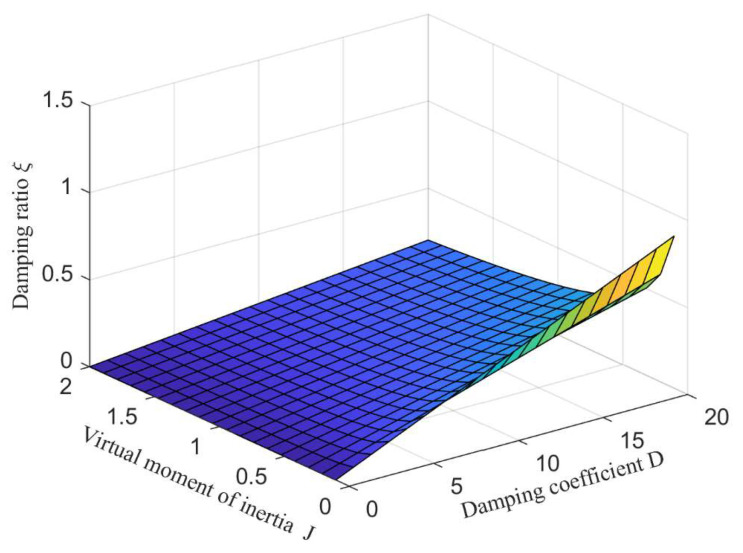
Three-dimensional relationship between virtual rotational inertia, damping coefficient and damping ratio.

Therefore, in the design of VSG parameters, the sag coefficient should be determined initially. Subsequently, the values of J, D, and $k_{f}$ are derived based on Equation (11). To achieve optimal system parameters while considering the system’s frequency modulation capability, the values of these three parameters must satisfy the specified constraints. When $k_{f}$ remains constant, Figure 9 illustrates the three-dimensional relationship between J, D, and ξ. When *D* is a fixed value, *J* increases, the damping ratio *ξ* decreases, the stability of the system becomes worse, and the power oscillation is more serious. *J* cannot be too large or too small. When *J* takes a fixed value, *D* increases, the damping ratio *ξ* increases, the system stability is enhanced, the power oscillation is suppressed, but the system response speed is slowed down. Through the adjustment of adaptive control, the values of *J* and *D* can be reasonably selected, so that the system can always work in the optimal damping ratio state.

## 4. Parameter-Adaptive VSG Control Strategy Design Approach

### 4.1. Influence of the Virtual Moment of Inertia and Damping Coefficient on the Stability of the System

J, D, and $k_{f}$ significantly influence the system’s dynamic performance. $k_{f}$ is typically set to a constant value. The relationship between variations in J and D and the system’s dynamic performance indices is presented in the Table 1.

### 4.2. System Frequency Comparison under Different Parameters

**Figure 10:**
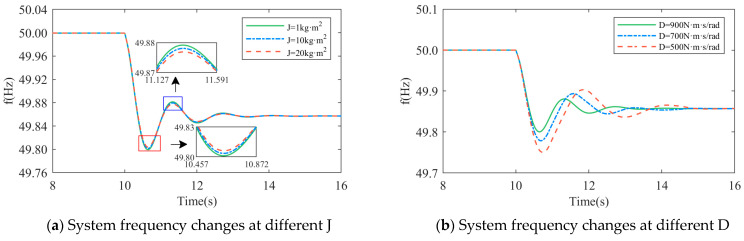
Comparison of system frequency changes under different *J* and *D*.

As shown in Figure 10a,b, the D-DPMSG-VSG system suddenly increases the load at t = 10 s, and the system frequency is compared under different parameters.

When *J* takes different values, D=900 N⋅m⋅s/rad; when D takes different values, $J=5\;\mathrm{kg}\cdot m^{2}$. It can be seen from the diagram that when *D* takes a fixed value and *J* changes within a certain range, the overshoot and adjustment time decrease with the increase of *J*; when *J* takes a fixed value, the overshoot decreases with the increase of *D*, but too large of a *D* will affect the response speed of the system, so the values of *J* and *D* should be reasonably selected. At the same time, it can also be seen that the change of *J* values mainly affects the rate of frequency change, and the change of *D* values mainly affect the lowest point of the frequency drop. The specific theoretical analysis is shown in Section 4.3.

### 4.3. Transient Process Analysis of the D-DPMSG-VSG System under a Load Disturbance

**Figure 11:**
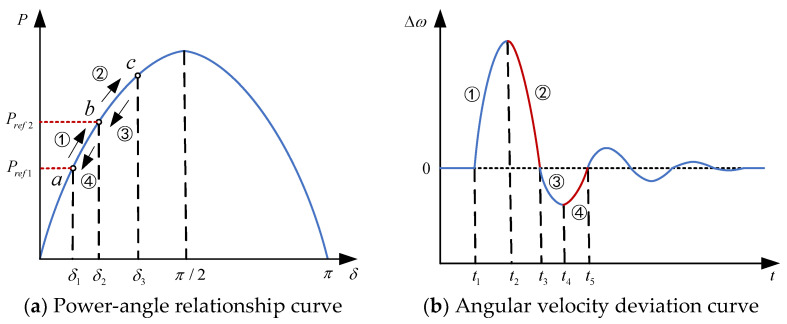
Power-angle relationship and angular velocity deviation curve.

When power fluctuations arise from sudden load changes during system operation, attenuation oscillations, similar to those observed in traditional synchronous generators, will occur, as illustrated in Figure 11.

For simplification, the transient decay oscillation process of the system is divided into four phases, with both J and D of the VSG held constant: Phase ①, $t_{1}-t_{2}$; Phase ②, $t_{2}-t_{3}$; Phase ③, $t_{3}-t_{4}$; and Phase ④, $t_{4}-t_{5}$. The system’s operating point change process is described by a→b→c→b→a. When the electromagnetic power satisfies $P<P_{ref2}$, the angular velocity ω accelerates, which is termed acceleration torque; when $P>P_{ref2}$, ω begins to decelerate, termed deceleration torque. In Phase ①, the angular velocity deviation of the VSG increases monotonically, with the virtual rotor angular velocity ω exceeding the rated velocity $\omega_{0}$. The angular velocity deviation Δω>0, and the rate of change of the angular velocity dω/dt>0. It can be determined that Δω(dω/dt)>0. To suppress these changes, J and D must be appropriately increased. In Phase ②, Δω>0 and dω/dt<0, and ω decreases monotonically but remains above the rated value. To accelerate the decay and stabilize at the rated value faster, J must be appropriately decreased, while D should be increased. The selection principles for J and D in Phases ③ and ④ are analogous to those in Phases ① and ②, and thus will not be reiterated.

The motion equation of the generator rotor is as follows:

(12) $$\left\{\begin{array}{l} P_{m}-P_{e}-D\Delta \omega =J\omega_{0}\frac{d\omega }{dt}\\ \Delta T-D\Delta \omega =J\frac{d\omega }{dt}\to D\Delta \omega =\Delta T-J\frac{d\omega }{dt} \end{array}\right.$$

It can be seen from Equation (12) that when ΔT−DΔω is constant, *J* is inversely proportional to dω/dt. When the virtual inertia *J* is larger, the inertia of VSG is larger, and the rate of angular velocity change dω/dt is smaller; when ΔT−J(dω/dt) is constant, *D* is inversely proportional to Δω. When *D* is greater, the damping effect of the system is greater, and the angular velocity deviation is smaller. At the same time, the increase in *D* will affect the initial value of the angular velocity change rate dω/dt.

In summary, the selection principles of *J* and *D* in this paper at different stages of the transient process are as follows.

The VSG parameter-adaptive control strategy, formulated on the basis of the parameter selection principles outlined in Table 2, is expressed as follows:

(13) $$\begin{array}{l} J=\left\{\begin{array}{l} J_{0},\Delta \omega (\frac{d\omega }{dt})\leq 0\cup \left|\frac{d\omega }{dt}\right|\leq T_{J}\\ J_{0}+k_{J}\left|\frac{d\omega }{dt}\right|,\Delta \omega (\frac{d\omega }{dt})>0\cap \left|\frac{d\omega }{dt}\right|>T_{J} \end{array}\right.\\ D=\left\{\begin{array}{l} D_{0},\left|\Delta \omega \right|\leq T_{D}\\ D_{0}+k_{D}\left|\Delta \omega \right|,\left|\Delta \omega \right|\geq T_{D} \end{array}\right. \end{array}$$

In Equation (13), J and D denote the adaptive virtual inertia and damping coefficients, respectively; $J_{0}$ and $D_{0}$ represent the steady-state values of inertia and damping, respectively; $k_{J}$ and $k_{d}$ are the adjustment factor for inertia and damping, respectively; $T_{J}$ and $T_{D}$ indicate the thresholds for the absolute rate of change of angular velocity and the absolute deviation of angular velocity, respectively. In the case of a frequency upward disturbance, when dω/dt is greater than 0, the frequency is greater than the rated value of 50 Hz and will continue to increase. The parameter-adaptive control proposed in this paper increases the *J* value to make $\left|d\omega /dt\right|$ smaller, and appropriately increases the *D* value to reduce the frequency deviation value, so that the speed of the frequency increase decreases; when dω/dt is less than 0, the frequency is greater than the rated value, but the speed of the upward disturbance of the frequency decreases. At this time, the parameter-adaptive control reduces the *J* value to make $\left|d\omega /dt\right|$ larger, which makes the speed of the frequency increase smaller and increases the *D* value, which not only reduces the frequency deviation value, but also increase the initial value of $\left|d\omega /dt\right|$, which makes the degree of the frequency disturbance smaller and suppresses the trend of the frequency increase. In the case of a downward frequency disturbance, the analysis process is similar to the previous analysis. The parameter-adaptive control proposed in this paper can make the frequency drop shallower and reduce the frequency drop speed when the frequency is disturbed downward. In summary, regardless of whether the system is in the process of an upward or downward disturbance, the parameter-adaptive control proposed in this paper can effectively suppress the overshoot when the frequency changes, which is helpful for maintaining the frequency stability. In some of the articles mentioned in the Section 1, some scholars will significantly increase or decrease the *D* value. However, by analyzing the simulation diagram of the fixed *J* value and a change of the *D* value in Section 4.2, we find that an increase of the *D* value has a significant effect on suppressing the lowest point of the frequency drop. Similarly, in the case of frequency disturbances, an increase of the *D* value has a very obvious effect on suppressing the highest point of the frequency rise. Therefore, in the adaptive regulation control proposed in this paper, the *D* value has been appropriately increased in the dynamic process of frequency change. Choosing an appropriate damping value adjustment coefficient $k_{d}$ will not sacrifice the system response speed too much.

**Figure 12:**
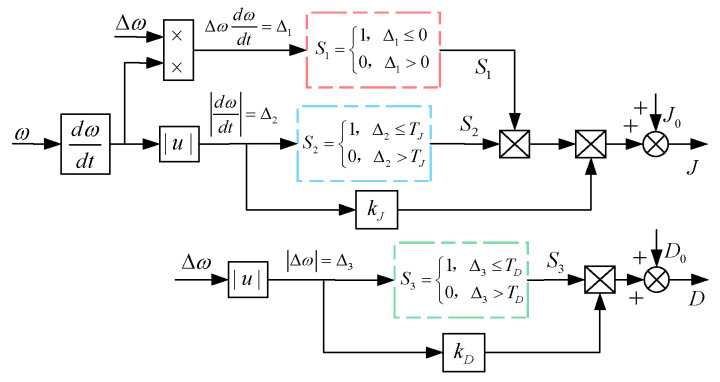
Block diagram of adaptive parameter control.

**Figure 13:**
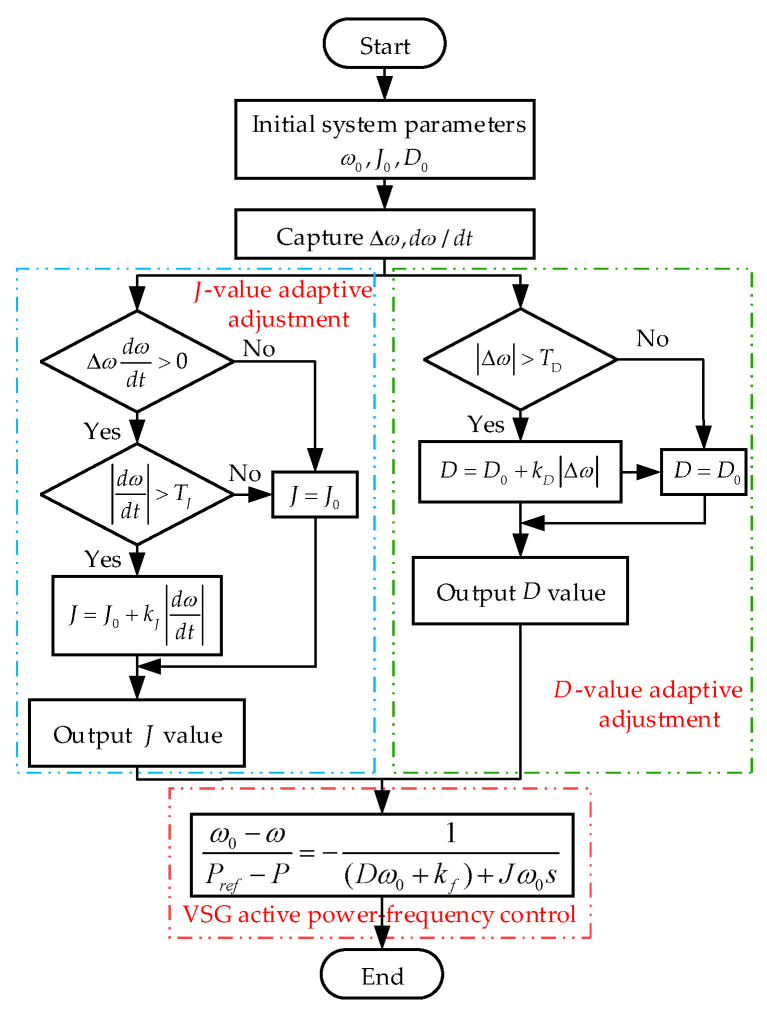
Parameter-adaptive flowchart.

The adaptive *J* and *D* control block diagrams are illustrated in Figure 12. The improved VSG parameter-adaptive control flowchart presented in this study is presented in Figure 13.

For this wind power system, $J_{0}=5\;\mathrm{kg}\cdot m^{2}$, $D_{0}=500\;N\cdot m\cdot s/\mathrm{rad}$, $k_{J}=0.5$, $k_{D}=200$, $T_{J}=1$, and $T_{D}=0.1$. First, the initial values for $\omega_{0}$, $J_{0}$ and $D_{0}$ are provided, followed by the acquisition of Δω and dω/dt values. If Δω(dω/dt)>0, the control parameter J is subjected to adaptive adjustment; if $\left|\Delta \omega \right|>T_{D}$, the control parameter D undergoes adaptive adjustment. Otherwise, J and D remain unchanged. The adjusted parameters are then fed into the active power frequency control loop of the VSG. Through this adaptive control, J and D of the VSG are dynamically regulated in response to frequency fluctuations, enabling the control parameters to respond to variations in the angular frequency deviation and its rate of change.

## 5. System Simulation and Analysis

**Figure 14:**
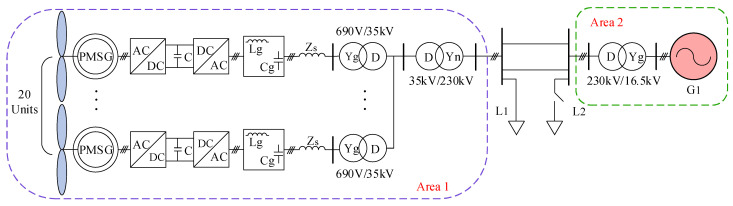
Two-machine, two-area grid simulation model.

In order to substantiate the efficacy and advantages of the adaptive control strategy delineated in this study, a two-machine, two-area simulation model was developed using simulation software, as depicted in Figure 14. The structure of a single grid-forming direct-drive permanent magnet wind turbine is illustrated in Figure 3.

In Figure 14, $L_{1}$ represents the initial load of the system, while $L_{2}$ denotes a load increase occurring suddenly. During the simulation, the active power of $L_{2}$ is increased to replicate a step load disturbance. $G_{1}$ is a synchronous generator with a rated capacity of 100 MW, an inertia time constant of 4 s, and a frequency modulation deadband of 0.033 Hz. Region 1 consists of a wind farm comprising 20 grid-forming permanent magnet wind turbines, each with a capacity of 2.5 MW. The system’s total installed capacity is 150 MW (1.0 pu), with a wind power penetration rate of 33.3%. The simulation parameters for the system are detailed in Table 3.

The verification of the improved parameter-adaptive VSG control participating in the system frequency modulation strategy is as follows: The total simulation time of the system is 16 s, and the load disturbance is added at 10 s. The simulation is divided into three working conditions. In working condition 1, the simulation of different load disturbances are under the same constant wind speed condition; in condition 2, the simulation of the same load disturbance is under different constant wind speed conditions. The load disturbance is set to 0.1 pu and 0.15 pu. In condition 3, under a turbulent wind speed with different average wind speeds, the load disturbance events occur in a period of gradually strong/weak gusts. The control strategies compared are MPPT control, VSG fixed parameter control (VSG-FPC), and VSG parameter-adaptive control (VSG-PAC. Among them, condition 1 is shown in subsection (1) below, condition 2 is shown in subsection (2) below, and condition 3 is shown in subsection (3) below.

**Figure 15:**
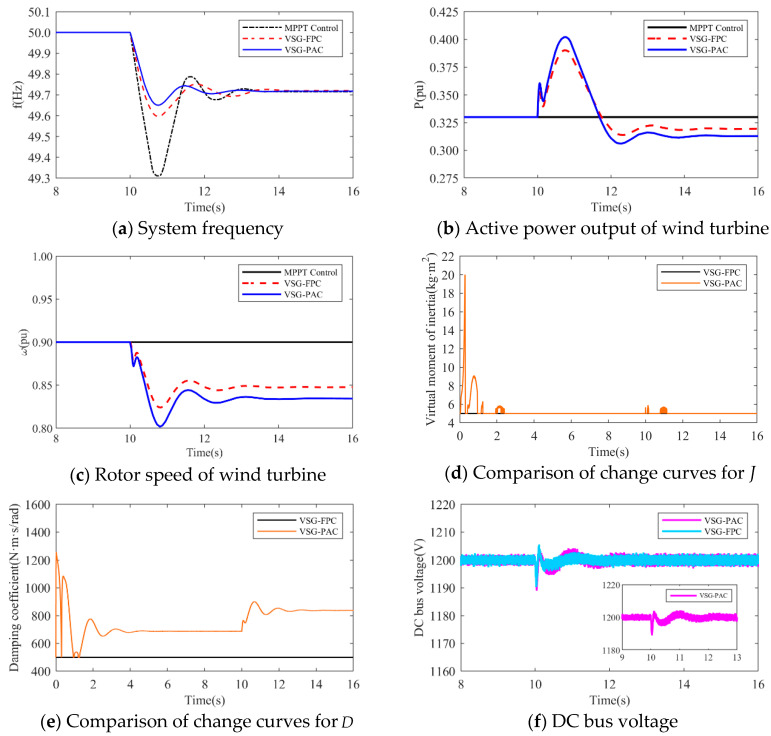
Simulated waveforms under load perturbation of 0.1 pu.

**Figure 16:**
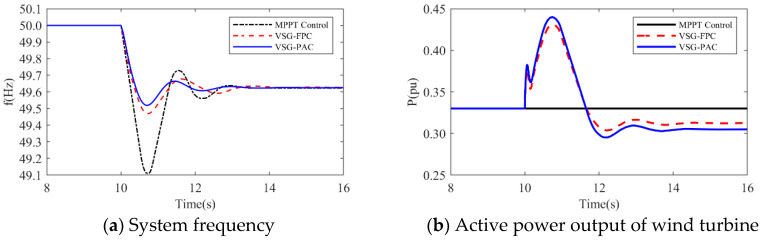

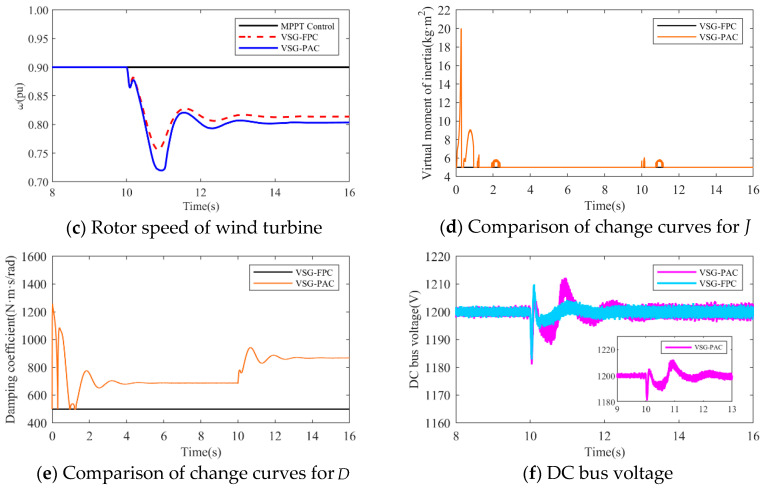
Simulated waveforms under load perturbation of 0.15 pu.

(1) The simulation wind speed is set at 8 m/s, and the wind turbine works in the maximum power tracking area. The simulation waveforms under two different load disturbance conditions are illustrated in Figure 15 and Figure 16. The simulation waveform display includes: a comparison of system frequency, active power output, and rotor speed of the wind turbine; curves of J and D; and the fluctuation of the DC bus voltage. Table 4 presents the system frequency data for the wind power system under various control strategies and different load disturbance conditions. To validate the effectiveness of the proposed VSG-PAC in enhancing the frequency regulation capability of wind turbines, a load disturbance of 0.1 pu and 0.15 pu is introduced at 10 s. The comparison control strategies are set as MPPT control and VSG-FPC.

Where $f_{L}$ denotes the value of the lowest frequency point,$f_{s}$ represents the steady-state frequency value, $\Delta f_{m}$ indicates the maximum frequency deviation, and $\Delta f_{s}$ signifies the steady-state frequency deviation. Analysis of Figure 15 and Figure 16, along with Table 4, reveals that: The frequency waveform simulation results demonstrate that incorporating VSG control into frequency modulation significantly enhances the wind turbine’s frequency support capability compared to traditional control methods.

**Figure 17:**
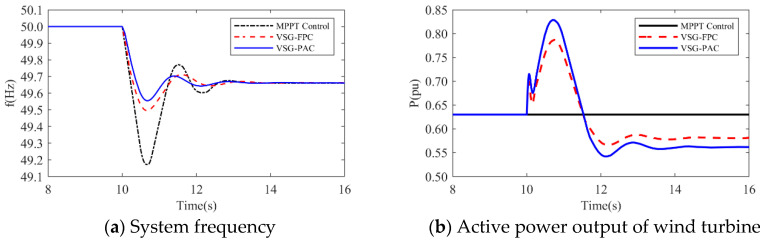

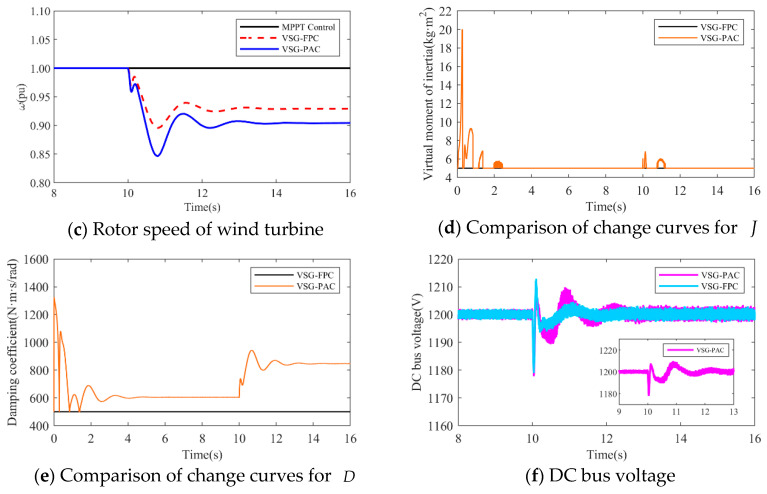
Simulated waveforms under load perturbation of 0.15 pu.

**Figure 18:**
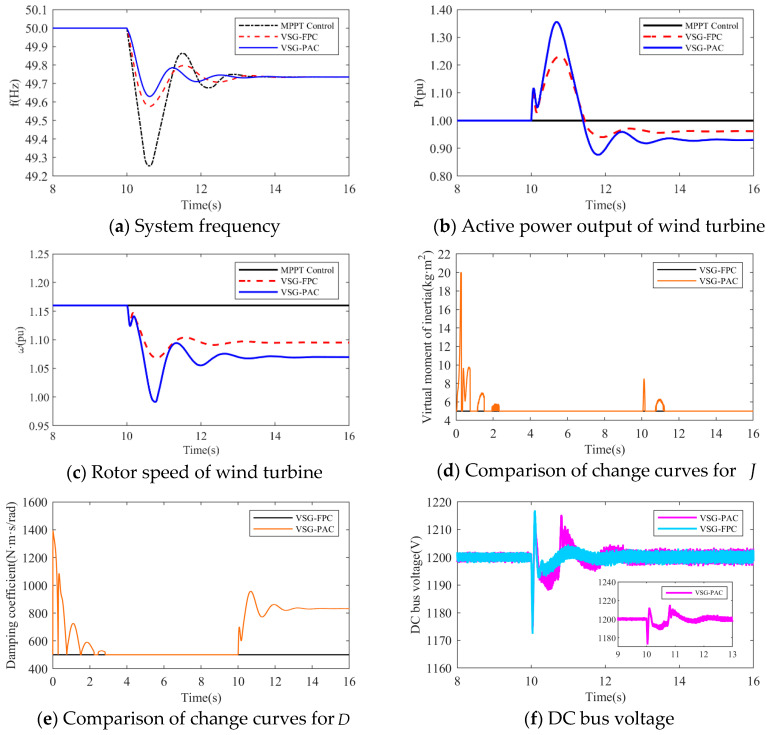
Simulated waveforms under load perturbation of 0.15 pu.

**Figure 19:**
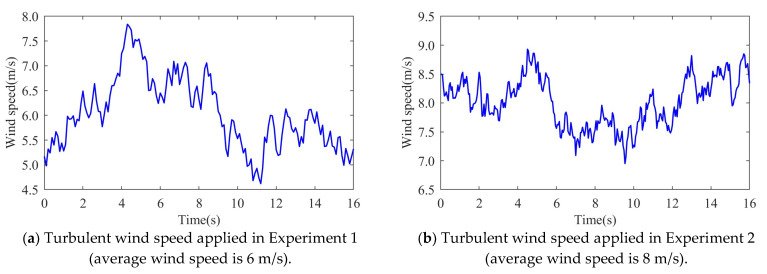
Turbulent wind speed.

1. When the load disturbance is 0.11 pu, the absence of an inertial response capability in MPPT control results in a rapid drop in the system frequency due to the load mutation. The lowest frequency observed is 49.3116 Hz, representing a significant drop. In comparison to the MPPT-controlled wind turbine, the wind turbine employing VSG-FPC exhibits some degree of inertial response and primary frequency modulation capabilities. VSG-FPC mitigates the rate of the system frequency change during the initial phase and offers frequency modulation power support during frequency drops, resulting in the lowest frequency drop point of 49.5967 Hz, an improvement of 0.2851 Hz. The enhanced VSG-PAC proposed in this paper further augments the frequency modulation capability of wind turbines, achieving the lowest frequency drop point of 49.6504 Hz. Compared to VSG-FPC, the maximum frequency deviation of the system increases by 13.3% with VSG-PAC. As illustrated in Figure 15a,b, with VSG-FPC and VSG-PAC, the frequency modulation active power output of the wind turbine rises from an initial value of 0.33 pu to peak values of 0.39 pu and 0.4 pu, respectively, while the rotor speed declines from an initial value of 0.9 pu to minimum values of 0.82 pu and 0.8 pu, respectively. Compared to VSG-FPC, VSG-PAC achieves a 2.6% increase in maximum active power output and a 2.5% reduction in the minimum rotor speed decrease. These results indicate that the improved VSG-PAC presented in this paper enables the wind turbine to release rotor kinetic energy more effectively, providing greater active support during frequency modulation and demonstrating clear advantages in frequency modulation performance. As illustrated in Figure 15f, the fluctuation in DC bus voltage is more pronounced with VSG-PAC compared to VSG-FPC. This is attributed to the fact that VSG-PAC enhances the wind turbines’ capacity to mitigate system frequency drops and increases the active power transmitted to the grid, resulting in greater instantaneous unbalanced power on the DC bus, thus widening the fluctuation range. However, the fluctuation range remains within permissible limits and will not adversely affect the DC bus equipment. The analysis under a 0.15 pu load disturbance condition yields similar results. For brevity, only a summary analysis is provided below.
2. Under a load disturbance of 0.15 pu, the lowest frequency drops of the system are 49.1102 Hz, 49.4689 Hz, and 49.5186 Hz, respectively, for MPPT, VSG-FPC, and VSG-PAC control. Compared to VSG-FPC, the maximum frequency deviation with VSG-PAC is increased by 9.4%. As illustrated in Figure 16a, compared to VSG-FPC, the output value of FM active power with VSG-PAC is improved only little. This occurs because, at this moment, the lowest rotor speed drop of the wind turbine is 0.719 pu, which is very close to 0.7 pu. The safety margin for the rotor speed is less than 0.02 pu, posing a risk of instability and turbine shutdown. In accordance with the established speed safety limits, the rotor speed cannot decrease further, thereby constraining the amount of released rotor kinetic energy. The improved VSG-PAC proposed in this paper offers substantial power support to the system while maintaining the safe operation of the wind turbine.
(2) The simulated wind speed is set to 11 m/s, with the wind turbine operating in a constant speed region. The simulated waveforms under a load disturbance condition of 0.15 pu are illustrated in the Figure 17.
  The simulated wind speed is set to 13 m/s, with the wind turbine operating in the constant power region. Figure 18 presents the simulated waveforms under a load disturbance condition of 0.15 pu. Table 5 illustrates the impact of different wind speeds on the system’s frequency support effectiveness under a 0.15 pu load disturbance. An analysis of Figure 17 and Figure 18 and Table 5 is shown below:

1. At a wind speed of 11 m/s, the minimum frequency drops of the system are 49.1722 Hz, 49.4957 Hz, and 49.555 Hz for MPPT, VSG-FPC, and VSG-PAC control, respectively. Compared to VSG-FPC, VSG-PAC exhibits an increase in the maximum frequency deviation of 11.8%. The enhanced control strategy proposed in this paper significantly improves the inertial support capability of VSG control and further reduces the maximum frequency deviation.
2. At a wind speed of 13 m/s, the minimum frequency drops of the system are 49.2552 Hz, 49.5761 Hz, and 49.6302 Hz for MPPT, VSG-FPC, and VSG-PAC control, respectively. Relative to VSG-FPC and MPPT control, VSG-PAC shows an increase in the maximum frequency deviation of 12.76% and 50%, respectively. As illustrated by the active power output and rotor speed waveforms of the wind turbine in Figure 18b,c, the enhanced control strategy presented in this paper effectively releases rotor kinetic energy during the inertia response phase and offers greater active support to the system.
(3) Taking the average annual wind speed of the Kubuqi Desert in Inner Mongolia as an example, the average annual wind speed at a 120 m height in this area is 6–8 m/s, and the average wind speed at a 160 height is 6.5–8.5 m/s. In order to verify the effectiveness of the proposed method in the turbulent wind speed scenario, the following two experimental scenarios are set up. As shown in Figure 19, in Experiment 1, the average wind speed is 6 m/s, and the turbulence degree is 25.6%. When t = 10 s, a sudden increase of a 0.02 pu load occurs during the weakening gust period. In Experiment 2, the average wind speed is 8 m/s, the turbulence degree is 10.6%, and the sudden load is 0.07 pu. The sudden load disturbance event occurs during the gradual gust period.

**Figure 20:**
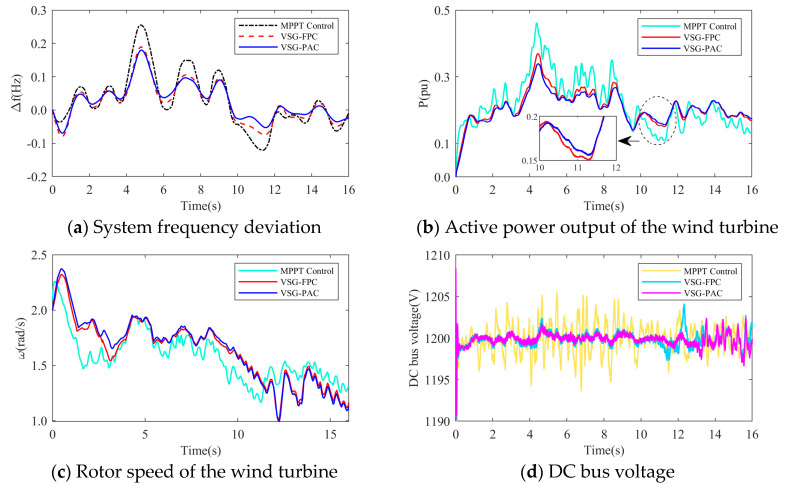

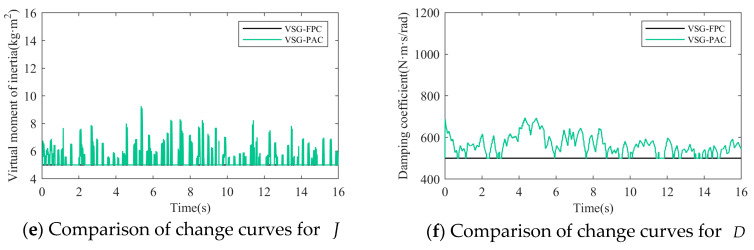
The simulation waveform of Experiment 1.

**Figure 21:**
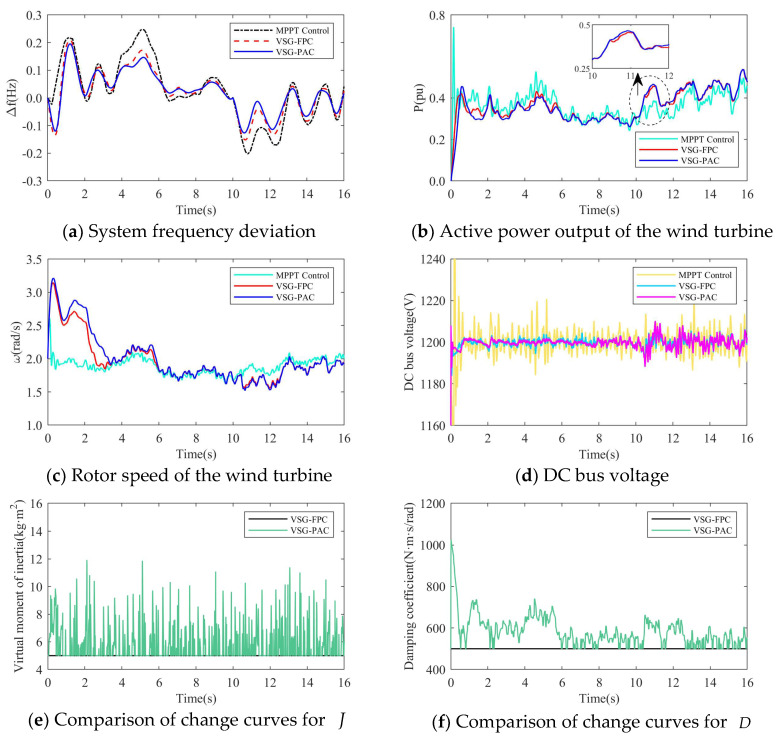
The simulation waveform of Experiment 2.

It should be noted that in Experiment 2, the wind turbine participates in frequency modulation under gradually strong gusts, and sufficient input aerodynamic power is conducive to maintaining the stability of the wind turbine; in Experiment 1, due to the superposition of the wind speed drop while the wind turbine releases the kinetic energy of the wind turbine, in order to ensure the operation stability of the wind turbine during the frequency modulation process, a smaller load disturbance is set in the simulation. The turbulent wind speed used in Experiment 1 and Experiment 2 was generated by Turbsim and IECwind, which met the IEC standard, as shown in the following figure. Figure 20 and Figure 21 are the simulation waveforms of the system under the conditions of Experiment 1 and Experiment 2, respectively.

In the above diagram, the frequency modulation effect comparison, wind power output comparison, rotor speed comparison, DC bus voltage comparison, and adaptive change curve comparison of *J* and *D* in the whole simulation process of wind turbines under different control strategies are compared. From Figure 20a and Figure 21a, it can be seen that with the change of turbulent wind speed, the system frequency also fluctuates. Compared with MPPT and VSG-FPC control, the improved VSG control in this paper can more effectively suppress frequency overshoot. When the system load increases suddenly at 10 s, the grid frequency of the three control strategies decreases, but the improved VSG control in this paper makes the decrease of the grid frequency significantly smaller than the other two control strategies. It can be seen from Figure 20b and Figure 21b that when the system load increases suddenly at 10 s, VSG-PAC can make the wind turbine output more power to support the primary frequency modulation of the power grid. At the same time, corresponding to Figure 20c and Figure 21c, VSG control can extract the rotor kinetic energy during the primary frequency modulation process. The active power is transmitted to support the grid frequency, so compared with the MPPT control, the VSG control has a significant drop in speed during the frequency modulation process. VSG-PAC makes the wind turbine extract more rotor kinetic energy, so the speed drop is greater. Figure 20d and Figure 21d show the change of DC bus voltage in the simulation process. Compared with MPPT control, VSG control can more effectively suppress the fluctuation of DC bus voltage, while VSG-PAC further suppresses the fluctuation of DC bus voltage compared with VSG-FPC under the normal operation of the wind turbine. After 10 s, the fluctuation of the DC bus voltage of VSG-PAC is slightly higher than that of VSG-FPC. As shown in the previous analysis using constant wind speed conditions, this is because VSG-PAC has a stronger ability to transmit active power in a short time under frequency modulation. At the same time, through Figure 20e,f and Figure 21e,f, it can also be seen that the parameters of *J* and *D* also change adaptively corresponding to the system frequency fluctuation. The change trend is consistent with the linear adaptive formula listed in Section 4, and the change trend of parameter adaptation will also be affected by the change trend of the turbulent wind speed. Compared with turbulent wind with an average wind speed of 6 m/s, the change amplitude and amplitude of the *J* value and *D* value are also larger under the condition of turbulent wind with an average wind speed of 8 m/s, which corresponds to the simulation results of Experiment 2, and the fluctuation of the system frequency is more intense.

## 6. Conclusions

In this paper, under the premise of considering the steady-state and dynamic characteristics of frequency modulation, the key parameters of voltage source VSG control, virtual moment of inertia *J*, and damping coefficient *D* are adaptively designed. In the two-machine, two-area power grid model, the frequency modulation effect of the proposed VSG parameter-adaptive control is verified by suddenly increasing the load disturbance under three constant wind speeds and two turbulent wind speeds. The main conclusions of theoretical analysis and simulation verification are as follows:

Firstly, the voltage source VSG control strategy endows inverters with port characteristics analogous to those of traditional synchronous machines, enabling them to actively emulate the inertia and frequency modulation functions of these traditional machines. This approach actively engages with variations in the grid-connected port to provide dynamic support for both port voltage and frequency. Secondly, the primary frequency regulation capability of the grid-forming direct-drive permanent magnet wind turbine is predominantly influenced by J, D, and $k_{f}$. Typically, $k_{f}$ is fixed. Given the constraints on these three parameters, J and D are adjusted adaptively, highlighting the flexible nature of VSG parameters. The adaptive VSG parameter control proposed herein offers superior frequency support compared to MPPT control and VSG constant parameter control, allowing for a more significant decrease in rotor speed without surpassing the limit, thereby substantially enhancing active support capabilities. The improved control strategy presented in this paper fully exploits the potential of rotor kinetic energy for frequency modulation in wind turbines, demonstrating significant advantages in providing short-term power increases to decelerate frequency changes and mitigate the depth of frequency drops. The scheme proposed in this paper offers valuable insights for the development of frequency modulation strategies in wind turbine systems.

## Figures and Tables

**Table 1:** Impact of J and D on system stability.

Indices	J No Change, D Increase	D No Change, J Increase
σ%	Diminish	Amplify
$t_{s}$	Faster	Slower
$t_{p}$	Faster	Slower
N	Diminish	Amplify

**Table 2:** Principles of selection of parameters for various stages.

Section	Δω	dω/dt	Δω(dω/dt)	J	D
①	>0	>0	>0	Amplify	Amplify
②	>0	<0	<0	Diminish	Amplify
③	<0	>0	<0	Amplify	Amplify
④	<0	<0	>0	Diminish	Amplify

**Table 3:** System Simulation Parameters.

**Wind turbine parameters**			
**Parameters**	**Value**	**Parameters**	**Value**
Rating power	2.5 MW	DC bus voltage	1200 V
Polar logarithm	80	Stator resistance	0.006 Ω
Stator inductance	0.395 H	Net-side filter inductor	0.0003 H
Net-side filter capacitors	25 μF	Rated frequency	50 Hz
**Synchronous generator parameters**			
**Parameters**	**Value**	**Parameters**	**Value**
Rated voltage	16.5 kV	Rated frequency	50 Hz
Rating power	100 MW	d-axis reactance	0.146 pu
q-axis reactance	0.0969 pu	d-axis transient reactance	0.0608 pu
q-axis sub-transient reactance	0.06 pu	q-axis sub-transient reactance	0.04 pu

**Table 4:** Frequency support effect under different load disturbance conditions.

Load Disturbances	Control Strategies	$f_{L}\left(\mathrm{Hz}\right)$	$f_{s}\left(\mathrm{Hz}\right)$	$\Delta f_{m}\left(\mathrm{Hz}\right)$	$\Delta f_{s}\left(\mathrm{Hz}\right)$
0.10 pu	MPPT control	49.3116	49.7153	−0.6884	−0.2847
	VSG-FPC	49.5967	49.721	−0.4033	−0.279
	VSG-PAC	49.6504	49.718	−0.3496	−0.282
0.15 pu	MPPT control	49.1102	49.623	−0.8898	−0.377
	VSG-FPC	49.4689	49.6279	−0.5311	−0.3721
	VSG-PAC	49.5186	49.6254	−0.4814	−0.3746

**Table 5:** Frequency support effect under different wind speed conditions.

Wind Speed	Control Strategies	$f_{L}\left(\mathrm{Hz}\right)$	$f_{s}\left(\mathrm{Hz}\right)$	$\Delta f_{m}\left(\mathrm{Hz}\right)$	$\Delta f_{s}\left(\mathrm{Hz}\right)$
8 m/s	MPPT control	49.1102	49.623	−0.8898	−0.377
	VSG-FPC	49.4689	49.6279	−0.5311	−0.3721
	VSG-PAC	49.5186	49.6254	−0.4814	−0.3746
11 m/s	MPPT control	49.1722	49.661	−0.8278	−0.339
	VSG-FPC	49.4957	49.6626	−0.5043	−0.3374
	VSG-PAC	49.555	49.6615	−0.445	−0.3385
13 m/s	MPPT control	49.2552	49.7355	−0.7448	−0.2645
	VSG-FPC	49.5761	49.7359	−0.4239	−0.2641
	VSG-PAC	49.6302	49.7356	−0.3698	−0.2644

## Data Availability

Data are contained within the article.
